# (*R*)-2,2′-Bis[*N*′-(3,5-dichloro­phen­yl)ureido]-1,1′-binaphthalene chloro­form disolvate

**DOI:** 10.1107/S1600536810052657

**Published:** 2011-01-15

**Authors:** Roman Holakovský, Michaela Pojarová, Michal Dušek, Jan Čejka, Ivana Císařová

**Affiliations:** a Department of Organic Chemistry, Institute of Chemical Technology Prague, Technická 5, 166 28 Prague 6, Czech Republic; b Institute of Physics, AS CR, v.v.i., Na Slovance 2, 182 21 Praha 8, Czech Republic; c Department of Solid State Chemistry, Institute of Chemical Technology Prague, Technická 5, 166 28 Prague 6, Czech Republic; d Charles University in Prague, Faculty of Science - Department of Inorganic Chemistry, Hlavova 2030/8, 128 43 Prague 2, Czech Republic

## Abstract

The title compound, C_34_H_22_Cl_4_N_4_O_2_·2CHCl_3_, is a new urea based on the 1,1′-binaphthalene skeleton, which crystallizes with two mol­ecules of binaphthalene and four mol­ecules of chloro­form in the unit cell. The chloro­form solvent mol­ecules do not participate in non-covalent inter­actions and therefore, can be found in several positions. The binaphthalene mol­ecules are connected *via* a system of N—H⋯O hydrogen bonds between the ureido units. C—H⋯O inter­actions also occur. In contrast to unsubstituted urea, where mol­ecules form squares in crystals, the bulky substituents disturb this arrangement and three ureido groups form infinite chains, while the fourth inter­acts with a neighbouring binaphthalene ring *via* an N—H⋯π inter­action. The solvent molecules are disordered with occupancy ratios of 0.60:0.40, 0.58:0.42, 0.50:0.50 and 0.77:0.23.

## Related literature

For background to 1,1′-binaphthalene derivatives and their use in mol­ecular recognition and catalysis, see: Pu (1998 ▶); Telfer & Kuroda (2003 ▶). For applications of urea derivatives based on the binaphthalene skeleton in chiral recognition, see: Stibor *et al.* (2004 ▶) and for their applications in the field of organocatalysis, see: Takemoto (2005 ▶); Fleming *et al.* (2006 ▶); Liu *et al.* (2007 ▶); Shi & Liu (2008 ▶); Harada *et al.* (2009 ▶). For the structure of urea, see: Sklar *et al.* (1961 ▶).
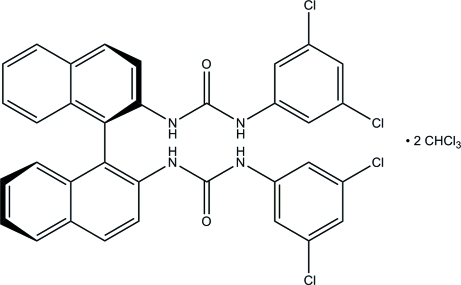


## Experimental

### 

#### Crystal data

C_34_H_22_Cl_4_N_4_O_2_·2CHCl_3_
*M* *_r_* = 899.09
Triclinic, 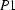

*a* = 11.879 (2) Å
*b* = 12.445 (3) Å
*c* = 15.039 (3) Å
α = 96.71 (3)°
β = 110.90 (3)°
γ = 103.98 (3)°
*V* = 1964.4 (9) Å^3^
*Z* = 2
Mo *K*α radiation
μ = 0.75 mm^−1^
*T* = 150 K
0.4 × 0.12 × 0.10 mm

#### Data collection

Bruker SMART 1000 diffractometer
20566 measured reflections
16629 independent reflections
13656 reflections with *I* > 2σ(*I*)
*R*_int_ = 0.037

#### Refinement

*R*[*F* ^2^ > 2σ(*F* ^2^)] = 0.050
*wR*(*F* ^2^) = 0.125
*S* = 1.03
16629 reflections
993 parameters
51 restraints
H-atom parameters constrained
Δρ_max_ = 0.63 e Å^−3^
Δρ_min_ = −0.58 e Å^−3^
Absolute structure: Flack (1983 ▶), 7535 Friedel pairs
Flack parameter: −0.01 (4)

### 

Data collection: *SMART* (Bruker, 2007 ▶); cell refinement: *SAINT* (Bruker, 2007 ▶); data reduction: *SAINT*; program(s) used to solve structure: *SHELXS97* (Sheldrick, 2008 ▶); program(s) used to refine structure: *SHELXL97* (Sheldrick, 2008 ▶); molecular graphics: *DIAMOND* (Brandenburg & Putz, 2005 ▶); software used to prepare material for publication: *publCIF* (Westrip, 2010 ▶).

## Supplementary Material

Crystal structure: contains datablocks I, global. DOI: 10.1107/S1600536810052657/om2383sup1.cif

Structure factors: contains datablocks I. DOI: 10.1107/S1600536810052657/om2383Isup2.hkl

Additional supplementary materials:  crystallographic information; 3D view; checkCIF report

## Figures and Tables

**Table 1:** Hydrogen-bond geometry (Å, °) *Cg*1 is the centroid of the C56–C61 ring.

*D*—H⋯*A*	*D*—H	H⋯*A*	*D*⋯*A*	*D*—H⋯*A*
N3—H3*A*⋯O4^i^	0.86	2.16	2.897 (3)	144
N4—H4*A*⋯O4^i^	0.86	2.05	2.821 (3)	149
N5—H5*A*⋯O2	0.86	2.11	2.821 (3)	139
N6—H6*A*⋯O2	0.86	2.17	2.947 (3)	150
N7—H7*A*⋯O3	0.86	2.36	2.955 (3)	126
N8—H8*A*⋯O3	0.86	2.26	3.030 (3)	149
C38—H38⋯O1^ii^	0.93	2.41	3.304 (4)	162
C54—H54⋯O4	0.93	2.32	2.893 (3)	120
C200—H200⋯O1^ii^	0.98	2.15	3.035 (7)	149
C300—H300⋯O3	0.98	2.52	3.315 (9)	138
N2—H2*A*⋯*Cg*1	0.86	2.59	3.300 (3)	141

Symmetry codes: (i) 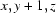; (ii) 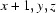.

**Fig. 1.**
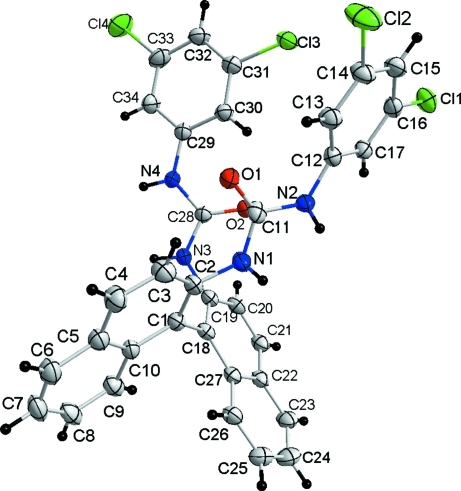
View of the one molecule of (R)-2,2'-bis[N-(3,5- dichlorophenyl)ureido]-1,1'-binaphthalene together with atom-labeling scheme. The hydrogen atoms which do not participate in hydrogen bonds and the solvents molecules were omitted for better clarity. Displacement ellipsoids are shown at the 50% probability level.

**Fig. 2.**
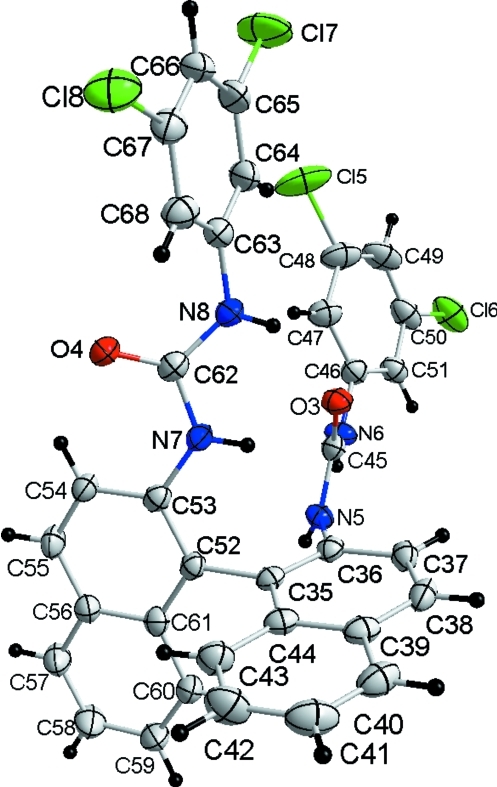
View of the other molecule of (R)-2,2'-bis[N-(3,5- dichlorophenyl)ureido]-1,1'-binaphthalene together with atom-labeling scheme. The hydrogen atoms which do not participate in hydrogen bonds and the solvents molecules were omitted for better clarity. Displacement ellipsoids are shown at the 50% probability level.

**Fig. 3.**
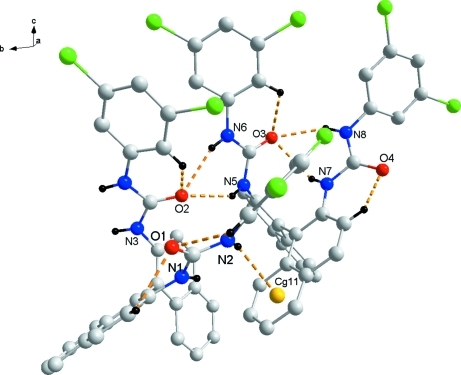
Projection in direction of the c axis with hydrogen bonds indicated.

## supplementary crystallographic information

### Comment

The title compound (Figs. 1 and 2) is a new urea based on 1,1'-binaphthalene skeleton. Because of their highly stable chiral configuration, the 2,2'-substituted 1,1'-binaphthyls have demonstrated outstanding chiral discrimination properties (Pu, 1998). Urea was prepared from (*R*)-1,1'-binaphthyl-2,2'-diamine by reaction with 3,5-dichlorophenyl isocyanate. Such molecule could be used as a ligand for chiral recognition of anions (Stibor *et al.*, 2004). The unit cell contains two molecules of substituted urea which are connected *via* system of intra- (N7—H7A···O3; N8—H8A···O3) and intermolecular hydrogen bonds (N3—H3A···O4; N4—H4A···O4; N5—H5A···O2; N6—H6A···O2) among three of four ureido moieties. Hydrogen bonds are listed in Table 1. The fourth ureido group interacts with the neighbouring binaphthalene ring in a N—H···π interaction (distance between N2—H2A and the center of aromatic ring C56-C61 is 2.587 (3) Å). In contrast to unsubstituted urea molecules, which form infinite straight chains perpendicular to each other in direction of *c* axis (Sklar *et al.*, 1961), the substitution with bulk substituents disturbs this arrangement and the hydrogen bonds form infinite wavy chains along the *b* axis (Fig. 3).

### Experimental

(*R*)-1,1'-Binaphthyl-2,2'-diamine (150 mg, 0.53 mmol) in dry dichloromethane (45 ml) was treated with 3,5-dichlorophenyl isocyanate (800 mg, 4,26 mmol, 4 eq per amino group) at ambient temperature for 12 h. The reaction was quenched with methanol (10 ml) and stirred for another 12 h. The reaction mixture was evaporated *in vacuo* and purified by column chromatography (silica gel, dichloromethane) to give the title compound as a white solid (97% yield). Single crystals of the title compound suitable for X-ray diffraction were obtained by slow evaporation of CDCl_3_ solution over a period of several days.

### Refinement

Four molecules of chloroform are present in the asymmetric unit. Solvent can freely rotate in its cavity which leads to disorder of its position. Two possible positions were found and refined for each molecule. The position of atoms were found from maps of electron densities, disordered fragments were then placed in appropriate positions, and all distances between neighbouring atoms and angles were fixed. Site occupancies were refined for the different parts with the same thermal parameters for the same atoms in the various fragments. At the end of the refinement, site occupancies were fixed. Hydrogen atoms were placed in calculated positions with N-H = 0.86 Å and C-H = 0.93 Å. Thermal parameters were set to U_iso_(H) equal to 1.2 times U_eq_ of the parent atom.

### Crystal data

**Table:**

C_34_H_22_Cl_4_N_4_O_2_·2CHCl_3_	*Z* = 2
*M**_r_* = 899.09	*F*(000) = 908
Triclinic, *P*1	*D*_x_ = 1.520 Mg m^−^^3^
Hall symbol: P 1	Mo *K*α radiation, λ = 0.71073 Å
*a* = 11.879 (2) Å	Cell parameters from 16629 reflections
*b* = 12.445 (3) Å	θ = 1.7–27.6°
*c* = 15.039 (3) Å	µ = 0.75 mm^−^^1^
α = 96.71 (3)°	*T* = 150 K
β = 110.90 (3)°	Plates, colourless
γ = 103.98 (3)°	0.4 × 0.12 × 0.10 mm
*V* = 1964.4 (9) Å^3^	

### Data collection

**Table:**

Bruker SMART 1000 diffractometer	13656 reflections with *I* > 2σ(*I*)
Radiation source: fine-focus sealed tube	*R*_int_ = 0.037
graphite	θ_max_ = 27.6°, θ_min_ = 1.7°
ω scans	*h* = −15→15
20566 measured reflections	*k* = −16→16
16629 independent reflections	*l* = −19→19

### Refinement

**Table:**

Refinement on *F*^2^	Secondary atom site location: difference Fourier map
Least-squares matrix: full	Hydrogen site location: inferred from neighbouring sites
*R*[*F*^2^ > 2σ(*F*^2^)] = 0.050	H-atom parameters constrained
*wR*(*F*^2^) = 0.125	*w* = 1/[σ^2^(*F*_o_^2^) + (0.0525*P*)^2^ + 1.6951*P*] where *P* = (*F*_o_^2^ + 2*F*_c_^2^)/3
*S* = 1.03	(Δ/σ)_max_ = 0.040
16629 reflections	Δρ_max_ = 0.63 e Å^−^^3^
993 parameters	Δρ_min_ = −0.58 e Å^−^^3^
51 restraints	Absolute structure: Flack (1983), 7535 Friedel pairs
0 constraints	Flack parameter: −0.01 (4)
Primary atom site location: structure-invariant direct methods	

### Special details

**Table:**

Geometry. All e.s.d.'s (except the e.s.d. in the dihedral angle between two l.s. planes) are estimated using the full covariance matrix. The cell e.s.d.'s are taken into account individually in the estimation of e.s.d.'s in distances, angles and torsion angles; correlations between e.s.d.'s in cell parameters are only used when they are defined by crystal symmetry. An approximate (isotropic) treatment of cell e.s.d.'s is used for estimating e.s.d.'s involving l.s. planes.
Refinement. Refinement of *F*^2^ against ALL reflections. The weighted *R*-factor *wR* and goodness of fit *S* are based on *F*^2^, conventional *R*-factors *R* are based on *F*, with *F* set to zero for negative *F*^2^. The threshold expression of *F*^2^ > σ(*F*^2^) is used only for calculating *R*-factors(gt) *etc*. and is not relevant to the choice of reflections for refinement. *R*-factors based on *F*^2^ are statistically about twice as large as those based on *F*, and *R*– factors based on ALL data will be even larger.

### Fractional atomic coordinates and isotropic or equivalent isotropic displacement parameters (Å^2^)

**Table:**

	*x*	*y*	*z*	*U*_iso_*/*U*_eq_	Occ. (<1)
Cl1	0.26222 (7)	0.20014 (6)	0.38434 (6)	0.04304 (19)	
Cl2	−0.18934 (8)	0.26670 (8)	0.31225 (7)	0.0613 (2)	
Cl3	0.19698 (7)	0.50494 (6)	0.49404 (5)	0.04183 (18)	
Cl4	0.37139 (10)	0.95394 (8)	0.65451 (6)	0.0624 (3)	
O1	0.00094 (17)	0.57167 (15)	0.16079 (14)	0.0331 (4)	
O2	0.35517 (17)	0.63877 (15)	0.25092 (13)	0.0295 (4)	
N1	0.0937 (2)	0.57038 (19)	0.05171 (17)	0.0331 (5)	
H1A	0.1373	0.5364	0.0307	0.040*	
N2	0.0982 (2)	0.43829 (19)	0.14486 (17)	0.0319 (5)	
H2A	0.1398	0.4156	0.1140	0.038*	
N3	0.3489 (2)	0.77884 (18)	0.16653 (15)	0.0269 (5)	
H3A	0.3421	0.8460	0.1661	0.032*	
N4	0.3253 (2)	0.80211 (18)	0.31075 (16)	0.0291 (5)	
H4A	0.3222	0.8683	0.3010	0.035*	
C1	0.1508 (2)	0.7245 (2)	−0.02232 (19)	0.0264 (6)	
C2	0.0655 (2)	0.6619 (2)	0.00973 (19)	0.0285 (6)	
C3	−0.0502 (3)	0.6840 (2)	−0.0042 (2)	0.0347 (7)	
H3	−0.1056	0.6425	0.0191	0.042*	
C4	−0.0803 (3)	0.7666 (3)	−0.0520 (2)	0.0377 (7)	
H4	−0.1563	0.7810	−0.0604	0.045*	
C5	0.0009 (3)	0.8303 (2)	−0.0887 (2)	0.0335 (7)	
C6	−0.0311 (3)	0.9125 (3)	−0.1431 (2)	0.0417 (8)	
H6	−0.1081	0.9260	−0.1542	0.050*	
C7	0.0474 (3)	0.9714 (3)	−0.1791 (2)	0.0447 (8)	
H7	0.0239	1.0241	−0.2151	0.054*	
C8	0.1652 (3)	0.9530 (2)	−0.1621 (2)	0.0407 (8)	
H8	0.2194	0.9942	−0.1865	0.049*	
C9	0.2002 (3)	0.8757 (2)	−0.1105 (2)	0.0350 (7)	
H9	0.2787	0.8655	−0.0990	0.042*	
C10	0.1188 (3)	0.8101 (2)	−0.07356 (19)	0.0291 (6)	
C11	0.0591 (2)	0.5303 (2)	0.1218 (2)	0.0291 (6)	
C12	0.0774 (2)	0.3766 (2)	0.2139 (2)	0.0282 (6)	
C13	−0.0334 (3)	0.3594 (2)	0.2302 (2)	0.0335 (6)	
H13	−0.0941	0.3932	0.1995	0.040*	
C14	−0.0502 (3)	0.2912 (3)	0.2930 (2)	0.0368 (7)	
C15	0.0374 (3)	0.2386 (3)	0.3409 (2)	0.0360 (7)	
H15	0.0235	0.1919	0.3823	0.043*	
C16	0.1472 (3)	0.2598 (2)	0.3232 (2)	0.0310 (6)	
C17	0.1677 (2)	0.3270 (2)	0.2606 (2)	0.0281 (6)	
H17	0.2417	0.3390	0.2499	0.034*	
C18	0.2721 (2)	0.6984 (2)	−0.00653 (18)	0.0245 (6)	
C19	0.3648 (2)	0.7205 (2)	0.08680 (19)	0.0277 (6)	
C20	0.4754 (2)	0.6888 (2)	0.1046 (2)	0.0299 (6)	
H20	0.5366	0.7053	0.1679	0.036*	
C21	0.4927 (2)	0.6340 (2)	0.0293 (2)	0.0307 (6)	
H21	0.5657	0.6127	0.0419	0.037*	
C22	0.4023 (2)	0.6087 (2)	−0.06752 (19)	0.0288 (6)	
C23	0.4167 (3)	0.5474 (3)	−0.1467 (2)	0.0374 (7)	
H23	0.4886	0.5245	−0.1351	0.045*	
C24	0.3276 (3)	0.5223 (3)	−0.2383 (2)	0.0435 (8)	
H24	0.3387	0.4825	−0.2893	0.052*	
C25	0.2184 (3)	0.5556 (3)	−0.2573 (2)	0.0362 (7)	
H25	0.1576	0.5375	−0.3209	0.043*	
C26	0.1997 (3)	0.6143 (2)	−0.1841 (2)	0.0322 (6)	
H26	0.1267	0.6360	−0.1982	0.039*	
C27	0.2910 (2)	0.6425 (2)	−0.08664 (19)	0.0275 (6)	
C28	0.3440 (2)	0.7334 (2)	0.24307 (18)	0.0241 (6)	
C29	0.3106 (2)	0.7745 (2)	0.39504 (19)	0.0267 (6)	
C30	0.2615 (2)	0.6638 (2)	0.40074 (19)	0.0283 (6)	
H30	0.2361	0.6035	0.3480	0.034*	
C31	0.2512 (2)	0.6454 (2)	0.4867 (2)	0.0305 (6)	
C32	0.2831 (3)	0.7321 (3)	0.5661 (2)	0.0346 (7)	
H32	0.2730	0.7178	0.6226	0.042*	
C33	0.3309 (3)	0.8411 (3)	0.5570 (2)	0.0400 (8)	
C34	0.3465 (3)	0.8651 (2)	0.4750 (2)	0.0326 (7)	
H34	0.3800	0.9397	0.4720	0.039*	
Cl5	0.57394 (13)	0.34425 (10)	0.69746 (8)	0.0873 (3)	
Cl6	0.60915 (9)	0.78573 (9)	0.73187 (6)	0.0607 (3)	
Cl7	0.73123 (14)	0.08571 (9)	0.71486 (7)	0.0854 (4)	
Cl8	0.69612 (8)	−0.20657 (7)	0.40606 (7)	0.0537 (2)	
O3	0.58803 (17)	0.40229 (15)	0.37528 (13)	0.0305 (4)	
O4	0.38945 (18)	0.01114 (15)	0.25737 (14)	0.0315 (5)	
N5	0.47199 (19)	0.46681 (18)	0.24838 (15)	0.0259 (5)	
H5A	0.4076	0.4909	0.2261	0.031*	
N6	0.4839 (2)	0.52764 (19)	0.40193 (16)	0.0320 (6)	
H6A	0.4352	0.5656	0.3741	0.038*	
N7	0.4394 (2)	0.18812 (18)	0.22683 (16)	0.0277 (5)	
H7A	0.5038	0.2464	0.2402	0.033*	
N8	0.5607 (2)	0.15267 (19)	0.36835 (16)	0.0308 (6)	
H8A	0.5976	0.2248	0.3857	0.037*	
C35	0.4606 (2)	0.3404 (2)	0.10734 (18)	0.0247 (6)	
C36	0.5279 (2)	0.4320 (2)	0.18452 (18)	0.0248 (6)	
C37	0.6511 (2)	0.4978 (2)	0.2005 (2)	0.0274 (6)	
H37	0.6955	0.5590	0.2540	0.033*	
C38	0.7056 (2)	0.4725 (2)	0.1385 (2)	0.0313 (6)	
H38	0.7868	0.5163	0.1497	0.038*	
C39	0.6394 (2)	0.3800 (2)	0.0573 (2)	0.0306 (6)	
C40	0.6942 (3)	0.3511 (3)	−0.0096 (2)	0.0384 (7)	
H40	0.7750	0.3947	0.0003	0.046*	
C41	0.6301 (3)	0.2610 (3)	−0.0869 (2)	0.0467 (8)	
H41	0.6669	0.2431	−0.1295	0.056*	
C42	0.5080 (3)	0.1948 (3)	−0.1025 (2)	0.0459 (8)	
H42	0.4648	0.1332	−0.1558	0.055*	
C43	0.4513 (3)	0.2185 (3)	−0.0415 (2)	0.0357 (7)	
H43	0.3702	0.1735	−0.0536	0.043*	
C44	0.5155 (2)	0.3116 (2)	0.04026 (19)	0.0282 (6)	
C45	0.5200 (2)	0.4621 (2)	0.34418 (19)	0.0257 (6)	
C46	0.5201 (2)	0.5380 (2)	0.5033 (2)	0.0316 (6)	
C47	0.5281 (3)	0.4448 (3)	0.5451 (2)	0.0400 (7)	
H47	0.5087	0.3732	0.5068	0.048*	
C48	0.5655 (3)	0.4610 (3)	0.6453 (2)	0.0496 (8)	
C49	0.5941 (3)	0.5648 (4)	0.7049 (2)	0.0521 (9)	
H49	0.6217	0.5743	0.7723	0.062*	
C50	0.5800 (3)	0.6546 (3)	0.6602 (2)	0.0431 (8)	
C51	0.5458 (2)	0.6426 (3)	0.5610 (2)	0.0328 (7)	
H51	0.5401	0.7050	0.5331	0.039*	
C52	0.3332 (2)	0.2647 (2)	0.09375 (18)	0.0242 (6)	
C53	0.3265 (2)	0.1840 (2)	0.14977 (18)	0.0257 (6)	
C54	0.2083 (2)	0.1063 (2)	0.1341 (2)	0.0293 (6)	
H54	0.2051	0.0504	0.1699	0.035*	
C55	0.1008 (3)	0.1138 (2)	0.0669 (2)	0.0326 (7)	
H55	0.0240	0.0622	0.0571	0.039*	
C56	0.1008 (3)	0.1980 (2)	0.0108 (2)	0.0301 (6)	
C57	−0.0113 (3)	0.2103 (3)	−0.0556 (2)	0.0394 (8)	
H57	−0.0890	0.1631	−0.0624	0.047*	
C58	−0.0077 (3)	0.2909 (3)	−0.1105 (2)	0.0457 (9)	
H58	−0.0825	0.2986	−0.1537	0.055*	
C59	0.1091 (3)	0.3610 (3)	−0.1008 (2)	0.0402 (8)	
H59	0.1112	0.4134	−0.1400	0.048*	
C60	0.2204 (3)	0.3545 (2)	−0.0352 (2)	0.0319 (7)	
H60	0.2969	0.4037	−0.0289	0.038*	
C61	0.2197 (2)	0.2725 (2)	0.02373 (19)	0.0272 (6)	
C62	0.4579 (2)	0.1102 (2)	0.28235 (19)	0.0274 (6)	
C63	0.6103 (3)	0.0830 (2)	0.4313 (2)	0.0318 (7)	
C64	0.6429 (3)	0.1150 (3)	0.5305 (2)	0.0409 (8)	
H64	0.6321	0.1813	0.5567	0.049*	
C65	0.6920 (3)	0.0463 (3)	0.5902 (2)	0.0459 (9)	
C66	0.7090 (3)	−0.0520 (3)	0.5542 (2)	0.0418 (8)	
H66	0.7421	−0.0969	0.5954	0.050*	
C67	0.6757 (3)	−0.0823 (2)	0.4553 (2)	0.0370 (7)	
C68	0.6273 (3)	−0.0162 (2)	0.3914 (2)	0.0357 (7)	
H68	0.6071	−0.0374	0.3249	0.043*	
Cl10	0.53490 (16)	0.01074 (15)	0.09971 (13)	0.0546 (4)	0.60
Cl12	0.79357 (19)	0.0613 (2)	0.11063 (18)	0.0980 (7)	0.60
Cl11	0.5872 (2)	−0.0818 (3)	−0.06273 (18)	0.0971 (8)	0.60
C100	0.6370 (5)	0.0287 (5)	0.0408 (4)	0.0498 (12)	0.60
H100	0.6287	0.0959	0.0141	0.060*	0.60
Cl13	0.5597 (3)	−0.0111 (3)	0.1169 (2)	0.0546 (4)	0.40
Cl14	0.7862 (4)	0.0531 (4)	0.0786 (3)	0.0971 (8)	0.40
Cl15	0.5513 (3)	−0.0905 (4)	−0.0699 (3)	0.0980 (7)	0.40
C101	0.6412 (7)	−0.0388 (8)	0.0563 (6)	0.0498 (12)	0.40
H101	0.6623	−0.1056	0.0774	0.060*	0.40
Cl20	0.82035 (18)	0.77629 (17)	0.17030 (17)	0.0558 (4)	0.58
Cl21	0.96509 (17)	0.73874 (16)	0.35521 (16)	0.0570 (3)	0.58
Cl22	1.0749 (2)	0.92854 (18)	0.2861 (2)	0.0697 (3)	0.58
C200	0.9738 (6)	0.7888 (5)	0.2535 (5)	0.0436 (9)	0.58
H200	1.0083	0.7401	0.2214	0.052*	0.58
Cl23	0.8182 (3)	0.7926 (3)	0.1869 (3)	0.0558 (4)	0.42
Cl25	1.0739 (4)	0.9381 (3)	0.2815 (4)	0.0697 (3)	0.42
Cl24	0.9869 (3)	0.7649 (3)	0.3707 (2)	0.0570 (3)	0.42
C201	0.9742 (9)	0.8007 (8)	0.2597 (7)	0.0436 (9)	0.42
H201	1.0011	0.7477	0.2245	0.052*	0.42
Cl30	0.9302 (4)	0.5635 (5)	0.5023 (3)	0.1098 (6)	0.50
Cl31	0.8503 (3)	0.4372 (3)	0.6273 (4)	0.1080 (6)	0.50
Cl32	0.8902 (3)	0.6767 (4)	0.6599 (3)	0.0959 (5)	0.50
C300	0.8422 (8)	0.5524 (7)	0.5724 (6)	0.0608 (11)	0.50
H300	0.7537	0.5393	0.5291	0.073*	0.50
Cl33	0.9259 (4)	0.5540 (6)	0.5012 (4)	0.1098 (6)	0.50
Cl35	0.8991 (4)	0.6884 (4)	0.6568 (3)	0.0959 (5)	0.50
Cl34	0.8638 (4)	0.4516 (4)	0.6438 (4)	0.1080 (6)	0.50
C301	0.8468 (9)	0.5568 (8)	0.5774 (7)	0.0608 (11)	0.50
H301	0.7567	0.5407	0.5370	0.073*	0.50
Cl40	1.01580 (19)	0.25846 (16)	0.58778 (16)	0.0958 (5)	0.77
Cl41	1.0437 (2)	0.03823 (16)	0.54162 (17)	0.1061 (7)	0.77
Cl42	1.22528 (18)	0.20149 (14)	0.71565 (12)	0.0890 (5)	0.77
C400	1.1179 (9)	0.1762 (6)	0.6000 (6)	0.134 (4)	0.77
H400	1.1683	0.2055	0.5638	0.161*	0.77
Cl45	1.2546 (6)	0.2198 (5)	0.6413 (4)	0.0890 (5)	0.23
C401	1.1031 (13)	0.1494 (12)	0.6348 (17)	0.134 (4)	0.23
H401	1.1074	0.1547	0.7015	0.161*	0.23
Cl44	1.0423 (9)	0.0034 (6)	0.5739 (6)	0.1061 (7)	0.23
Cl43	0.9875 (7)	0.2056 (6)	0.5716 (6)	0.0958 (5)	0.23

### Atomic displacement parameters (Å^2^)

**Table:**

	*U*^11^	*U*^22^	*U*^33^	*U*^12^	*U*^13^	*U*^23^
Cl1	0.0427 (4)	0.0395 (4)	0.0477 (4)	0.0165 (3)	0.0133 (3)	0.0198 (3)
Cl2	0.0496 (4)	0.0799 (5)	0.0815 (5)	0.0243 (4)	0.0457 (3)	0.0451 (4)
Cl3	0.0580 (4)	0.0343 (3)	0.0356 (3)	0.0058 (3)	0.0260 (3)	0.0109 (3)
Cl4	0.0915 (6)	0.0462 (5)	0.0404 (4)	0.0106 (4)	0.0293 (4)	−0.0117 (3)
O1	0.0415 (9)	0.0286 (9)	0.0408 (10)	0.0159 (7)	0.0249 (8)	0.0119 (7)
O2	0.0394 (9)	0.0275 (9)	0.0306 (9)	0.0168 (7)	0.0181 (7)	0.0121 (7)
N1	0.0406 (10)	0.0318 (11)	0.0442 (12)	0.0189 (9)	0.0285 (9)	0.0178 (9)
N2	0.0420 (11)	0.0303 (11)	0.0394 (11)	0.0171 (9)	0.0285 (9)	0.0141 (9)
N3	0.0360 (10)	0.0231 (10)	0.0264 (10)	0.0133 (8)	0.0143 (8)	0.0086 (8)
N4	0.0440 (11)	0.0229 (10)	0.0278 (10)	0.0145 (9)	0.0193 (9)	0.0068 (8)
C1	0.0282 (11)	0.0272 (12)	0.0254 (12)	0.0093 (10)	0.0112 (9)	0.0079 (9)
C2	0.0335 (12)	0.0275 (12)	0.0284 (12)	0.0125 (10)	0.0132 (10)	0.0115 (10)
C3	0.0298 (12)	0.0396 (15)	0.0393 (14)	0.0121 (11)	0.0161 (11)	0.0147 (12)
C4	0.0333 (13)	0.0417 (15)	0.0447 (16)	0.0194 (11)	0.0164 (12)	0.0145 (12)
C5	0.0397 (14)	0.0302 (13)	0.0298 (13)	0.0132 (11)	0.0110 (11)	0.0076 (11)
C6	0.0462 (15)	0.0392 (15)	0.0406 (16)	0.0223 (13)	0.0112 (13)	0.0128 (12)
C7	0.0562 (17)	0.0340 (14)	0.0431 (16)	0.0216 (13)	0.0111 (14)	0.0173 (12)
C8	0.0500 (17)	0.0290 (14)	0.0355 (15)	0.0063 (13)	0.0101 (13)	0.0139 (11)
C9	0.0381 (14)	0.0295 (13)	0.0334 (14)	0.0066 (11)	0.0114 (12)	0.0099 (11)
C10	0.0380 (13)	0.0235 (12)	0.0265 (12)	0.0124 (10)	0.0111 (10)	0.0071 (10)
C11	0.0253 (12)	0.0224 (12)	0.0383 (14)	0.0019 (10)	0.0150 (11)	0.0060 (10)
C12	0.0323 (12)	0.0220 (12)	0.0302 (12)	0.0047 (10)	0.0150 (10)	0.0050 (10)
C13	0.0326 (12)	0.0355 (14)	0.0392 (14)	0.0118 (11)	0.0196 (11)	0.0130 (11)
C14	0.0325 (12)	0.0400 (15)	0.0465 (15)	0.0093 (11)	0.0246 (11)	0.0172 (12)
C15	0.0404 (14)	0.0360 (15)	0.0321 (13)	0.0073 (12)	0.0171 (11)	0.0107 (11)
C16	0.0343 (13)	0.0261 (12)	0.0320 (13)	0.0092 (10)	0.0119 (11)	0.0083 (10)
C17	0.0276 (11)	0.0231 (12)	0.0345 (13)	0.0039 (10)	0.0164 (10)	0.0043 (10)
C18	0.0266 (11)	0.0240 (11)	0.0246 (11)	0.0071 (9)	0.0109 (9)	0.0111 (9)
C19	0.0340 (12)	0.0267 (12)	0.0272 (12)	0.0113 (10)	0.0142 (10)	0.0126 (9)
C20	0.0269 (12)	0.0332 (13)	0.0288 (12)	0.0096 (10)	0.0081 (10)	0.0128 (10)
C21	0.0317 (12)	0.0361 (13)	0.0336 (13)	0.0149 (10)	0.0179 (10)	0.0163 (10)
C22	0.0326 (12)	0.0292 (12)	0.0306 (12)	0.0106 (10)	0.0165 (10)	0.0137 (10)
C23	0.0454 (14)	0.0459 (16)	0.0358 (13)	0.0210 (12)	0.0265 (11)	0.0167 (11)
C24	0.0533 (16)	0.0521 (18)	0.0334 (14)	0.0181 (14)	0.0257 (13)	0.0086 (13)
C25	0.0359 (14)	0.0426 (16)	0.0267 (13)	0.0057 (12)	0.0129 (11)	0.0067 (11)
C26	0.0371 (13)	0.0336 (13)	0.0278 (13)	0.0126 (11)	0.0119 (11)	0.0131 (10)
C27	0.0336 (12)	0.0260 (12)	0.0263 (12)	0.0079 (10)	0.0148 (10)	0.0112 (9)
C28	0.0253 (11)	0.0241 (12)	0.0220 (11)	0.0078 (9)	0.0081 (9)	0.0053 (9)
C29	0.0289 (12)	0.0244 (12)	0.0272 (12)	0.0088 (10)	0.0111 (10)	0.0056 (9)
C30	0.0309 (12)	0.0294 (13)	0.0245 (12)	0.0080 (10)	0.0121 (10)	0.0052 (10)
C31	0.0305 (12)	0.0333 (13)	0.0294 (13)	0.0098 (11)	0.0128 (10)	0.0103 (10)
C32	0.0378 (13)	0.0385 (15)	0.0269 (13)	0.0070 (12)	0.0158 (11)	0.0051 (11)
C33	0.0456 (15)	0.0397 (16)	0.0288 (14)	0.0096 (13)	0.0137 (12)	−0.0030 (12)
C34	0.0341 (13)	0.0267 (13)	0.0332 (14)	0.0038 (11)	0.0138 (11)	0.0027 (11)
Cl5	0.1306 (8)	0.0989 (6)	0.0758 (5)	0.0560 (6)	0.0630 (5)	0.0646 (5)
Cl6	0.0593 (5)	0.0742 (6)	0.0334 (4)	0.0176 (4)	0.0106 (4)	−0.0131 (4)
Cl7	0.1381 (10)	0.0531 (5)	0.0294 (4)	0.0349 (6)	−0.0080 (5)	0.0004 (4)
Cl8	0.0665 (4)	0.0519 (4)	0.0599 (5)	0.0401 (3)	0.0278 (4)	0.0206 (3)
O3	0.0352 (9)	0.0273 (9)	0.0298 (9)	0.0135 (7)	0.0105 (7)	0.0089 (7)
O4	0.0350 (9)	0.0232 (9)	0.0330 (9)	0.0076 (7)	0.0095 (8)	0.0098 (7)
N5	0.0259 (10)	0.0267 (10)	0.0246 (10)	0.0112 (8)	0.0076 (8)	0.0052 (8)
N6	0.0411 (11)	0.0323 (11)	0.0248 (11)	0.0192 (10)	0.0101 (9)	0.0073 (9)
N7	0.0270 (10)	0.0223 (10)	0.0339 (11)	0.0065 (8)	0.0113 (9)	0.0117 (8)
N8	0.0356 (11)	0.0215 (10)	0.0306 (11)	0.0079 (9)	0.0079 (9)	0.0078 (9)
C35	0.0288 (11)	0.0218 (11)	0.0246 (11)	0.0096 (9)	0.0091 (9)	0.0104 (9)
C36	0.0270 (11)	0.0217 (11)	0.0278 (12)	0.0093 (9)	0.0107 (10)	0.0098 (9)
C37	0.0256 (11)	0.0208 (11)	0.0318 (13)	0.0051 (9)	0.0083 (10)	0.0052 (10)
C38	0.0234 (11)	0.0283 (12)	0.0450 (15)	0.0078 (10)	0.0141 (11)	0.0172 (11)
C39	0.0313 (12)	0.0378 (13)	0.0304 (12)	0.0172 (11)	0.0143 (10)	0.0159 (10)
C40	0.0389 (13)	0.0463 (16)	0.0460 (15)	0.0203 (12)	0.0263 (11)	0.0238 (12)
C41	0.0523 (15)	0.068 (2)	0.0382 (15)	0.0316 (15)	0.0278 (12)	0.0201 (14)
C42	0.0539 (17)	0.0534 (19)	0.0320 (15)	0.0177 (15)	0.0203 (13)	0.0025 (13)
C43	0.0403 (14)	0.0375 (15)	0.0324 (13)	0.0112 (12)	0.0188 (11)	0.0069 (11)
C44	0.0318 (12)	0.0304 (12)	0.0281 (12)	0.0149 (10)	0.0129 (10)	0.0127 (10)
C45	0.0283 (12)	0.0196 (11)	0.0273 (12)	0.0056 (10)	0.0100 (10)	0.0049 (9)
C46	0.0297 (12)	0.0375 (14)	0.0287 (13)	0.0087 (11)	0.0136 (10)	0.0086 (11)
C47	0.0442 (14)	0.0454 (16)	0.0392 (15)	0.0183 (13)	0.0212 (12)	0.0169 (12)
C48	0.0580 (17)	0.067 (2)	0.0463 (16)	0.0307 (15)	0.0325 (13)	0.0316 (14)
C49	0.0508 (16)	0.086 (2)	0.0282 (14)	0.0285 (17)	0.0198 (13)	0.0153 (15)
C50	0.0354 (14)	0.0590 (19)	0.0308 (15)	0.0142 (14)	0.0117 (12)	0.0001 (13)
C51	0.0297 (12)	0.0396 (15)	0.0286 (13)	0.0109 (11)	0.0115 (11)	0.0058 (11)
C52	0.0278 (11)	0.0189 (11)	0.0258 (12)	0.0060 (9)	0.0118 (9)	0.0036 (9)
C53	0.0298 (12)	0.0224 (11)	0.0267 (12)	0.0075 (10)	0.0138 (10)	0.0055 (9)
C54	0.0306 (12)	0.0236 (12)	0.0342 (13)	0.0054 (10)	0.0144 (10)	0.0097 (10)
C55	0.0234 (12)	0.0266 (13)	0.0412 (15)	0.0011 (10)	0.0105 (11)	0.0055 (11)
C56	0.0290 (12)	0.0256 (13)	0.0291 (13)	0.0046 (10)	0.0070 (11)	0.0045 (10)
C57	0.0316 (14)	0.0311 (14)	0.0439 (16)	0.0048 (12)	0.0048 (13)	0.0089 (12)
C58	0.0369 (15)	0.0399 (16)	0.0476 (18)	0.0103 (13)	0.0014 (14)	0.0160 (14)
C59	0.0444 (16)	0.0293 (14)	0.0357 (15)	0.0051 (12)	0.0056 (13)	0.0129 (12)
C60	0.0329 (13)	0.0260 (13)	0.0301 (13)	0.0036 (11)	0.0085 (11)	0.0071 (10)
C61	0.0315 (12)	0.0199 (11)	0.0271 (12)	0.0064 (10)	0.0104 (10)	0.0017 (9)
C62	0.0309 (12)	0.0252 (12)	0.0301 (12)	0.0099 (10)	0.0155 (10)	0.0080 (10)
C63	0.0267 (12)	0.0300 (13)	0.0328 (14)	0.0057 (11)	0.0062 (11)	0.0102 (11)
C64	0.0431 (16)	0.0262 (13)	0.0372 (16)	0.0064 (12)	0.0006 (13)	0.0061 (12)
C65	0.0482 (18)	0.0362 (16)	0.0325 (16)	0.0048 (14)	−0.0019 (14)	0.0066 (13)
C66	0.0406 (16)	0.0336 (15)	0.0365 (16)	0.0103 (13)	−0.0018 (13)	0.0118 (12)
C67	0.0299 (13)	0.0339 (14)	0.0480 (16)	0.0131 (11)	0.0130 (12)	0.0131 (12)
C68	0.0341 (13)	0.0370 (14)	0.0414 (15)	0.0152 (11)	0.0162 (11)	0.0156 (12)
Cl10	0.0575 (6)	0.0552 (8)	0.0611 (7)	0.0172 (6)	0.0338 (5)	0.0164 (6)
Cl12	0.0575 (7)	0.1097 (14)	0.1041 (12)	−0.0068 (9)	0.0473 (8)	−0.0403 (11)
Cl11	0.0871 (11)	0.1210 (17)	0.0700 (9)	0.0097 (12)	0.0447 (9)	−0.0237 (10)
C100	0.061 (2)	0.049 (3)	0.052 (3)	0.025 (2)	0.030 (2)	0.015 (2)
Cl13	0.0575 (6)	0.0552 (8)	0.0611 (7)	0.0172 (6)	0.0338 (5)	0.0164 (6)
Cl14	0.0871 (11)	0.1210 (17)	0.0700 (9)	0.0097 (12)	0.0447 (9)	−0.0237 (10)
Cl15	0.0575 (7)	0.1097 (14)	0.1041 (12)	−0.0068 (9)	0.0473 (8)	−0.0403 (11)
C101	0.061 (2)	0.049 (3)	0.052 (3)	0.025 (2)	0.030 (2)	0.015 (2)
Cl20	0.0459 (5)	0.0426 (6)	0.0719 (8)	0.0104 (4)	0.0206 (5)	0.0031 (5)
Cl21	0.0435 (5)	0.0539 (8)	0.0706 (6)	0.0065 (5)	0.0245 (5)	0.0143 (5)
Cl22	0.0542 (5)	0.0328 (5)	0.1189 (9)	0.0025 (4)	0.0384 (5)	0.0159 (5)
C200	0.0372 (14)	0.0277 (16)	0.074 (2)	0.0081 (13)	0.0324 (14)	0.0097 (15)
Cl23	0.0459 (5)	0.0426 (6)	0.0719 (8)	0.0104 (4)	0.0206 (5)	0.0031 (5)
Cl25	0.0542 (5)	0.0328 (5)	0.1189 (9)	0.0025 (4)	0.0384 (5)	0.0159 (5)
Cl24	0.0435 (5)	0.0539 (8)	0.0706 (6)	0.0065 (5)	0.0245 (5)	0.0143 (5)
C201	0.0372 (14)	0.0277 (16)	0.074 (2)	0.0081 (13)	0.0324 (14)	0.0097 (15)
Cl30	0.0853 (7)	0.1689 (16)	0.0845 (8)	0.0361 (9)	0.0492 (6)	0.0189 (9)
Cl31	0.0553 (7)	0.1114 (10)	0.1534 (14)	0.0252 (7)	0.0255 (8)	0.0680 (9)
Cl32	0.0726 (7)	0.1074 (10)	0.0802 (9)	0.0408 (7)	0.0007 (7)	−0.0124 (7)
C300	0.0396 (17)	0.076 (3)	0.062 (2)	0.0186 (17)	0.0159 (17)	0.0119 (19)
Cl33	0.0853 (7)	0.1689 (16)	0.0845 (8)	0.0361 (9)	0.0492 (6)	0.0189 (9)
Cl35	0.0726 (7)	0.1074 (10)	0.0802 (9)	0.0408 (7)	0.0007 (7)	−0.0124 (7)
Cl34	0.0553 (7)	0.1114 (10)	0.1534 (14)	0.0252 (7)	0.0255 (8)	0.0680 (9)
C301	0.0396 (17)	0.076 (3)	0.062 (2)	0.0186 (17)	0.0159 (17)	0.0119 (19)
Cl40	0.1081 (10)	0.0834 (12)	0.0976 (11)	0.0496 (9)	0.0318 (9)	0.0140 (10)
Cl41	0.1144 (12)	0.0655 (11)	0.1072 (16)	0.0192 (10)	0.0182 (12)	0.0061 (9)
Cl42	0.1057 (11)	0.0682 (8)	0.0712 (9)	0.0244 (8)	0.0100 (8)	0.0226 (7)
C400	0.167 (7)	0.098 (5)	0.084 (6)	0.060 (5)	−0.022 (5)	0.013 (4)
Cl45	0.1057 (11)	0.0682 (8)	0.0712 (9)	0.0244 (8)	0.0100 (8)	0.0226 (7)
C401	0.167 (7)	0.098 (5)	0.084 (6)	0.060 (5)	−0.022 (5)	0.013 (4)
Cl44	0.1144 (12)	0.0655 (11)	0.1072 (16)	0.0192 (10)	0.0182 (12)	0.0061 (9)
Cl43	0.1081 (10)	0.0834 (12)	0.0976 (11)	0.0496 (9)	0.0318 (9)	0.0140 (10)

### Geometric parameters (Å, °)

**Table:**

Cl1—C16	1.738 (3)	N8—C63	1.427 (4)
Cl2—C14	1.740 (3)	N8—H8A	0.8600
Cl3—C31	1.742 (3)	C35—C36	1.369 (3)
Cl4—C33	1.745 (3)	C35—C44	1.438 (4)
O1—C11	1.215 (4)	C35—C52	1.505 (4)
O2—C28	1.231 (3)	C36—C37	1.413 (4)
N1—C11	1.364 (4)	C37—C38	1.359 (4)
N1—C2	1.417 (4)	C37—H37	0.9300
N1—H1A	0.8600	C38—C39	1.408 (4)
N2—C11	1.372 (4)	C38—H38	0.9300
N2—C12	1.417 (4)	C39—C44	1.425 (4)
N2—H2A	0.8600	C39—C40	1.436 (4)
N3—C28	1.354 (3)	C40—C41	1.357 (4)
N3—C19	1.422 (4)	C40—H40	0.9300
N3—H3A	0.8600	C41—C42	1.404 (5)
N4—C28	1.366 (4)	C41—H41	0.9300
N4—C29	1.409 (4)	C42—C43	1.363 (5)
N4—H4A	0.8600	C42—H42	0.9300
C1—C2	1.386 (4)	C43—C44	1.417 (4)
C1—C10	1.432 (4)	C43—H43	0.9300
C1—C18	1.496 (4)	C46—C51	1.377 (4)
C2—C3	1.413 (4)	C46—C47	1.391 (4)
C3—C4	1.368 (4)	C47—C48	1.384 (5)
C3—H3	0.9300	C47—H47	0.9300
C4—C5	1.406 (4)	C48—C49	1.374 (5)
C4—H4	0.9300	C49—C50	1.384 (5)
C5—C6	1.422 (4)	C49—H49	0.9300
C5—C10	1.425 (4)	C50—C51	1.377 (4)
C6—C7	1.350 (5)	C51—H51	0.9300
C6—H6	0.9300	C52—C53	1.390 (4)
C7—C8	1.411 (5)	C52—C61	1.418 (4)
C7—H7	0.9300	C53—C54	1.420 (4)
C8—C9	1.355 (4)	C54—C55	1.349 (4)
C8—H8	0.9300	C54—H54	0.9300
C9—C10	1.420 (4)	C55—C56	1.420 (4)
C9—H9	0.9300	C55—H55	0.9300
C12—C17	1.380 (4)	C56—C57	1.406 (4)
C12—C13	1.395 (4)	C56—C61	1.422 (4)
C13—C14	1.375 (4)	C57—C58	1.374 (5)
C13—H13	0.9300	C57—H57	0.9300
C14—C15	1.392 (4)	C58—C59	1.396 (5)
C15—C16	1.391 (4)	C58—H58	0.9300
C15—H15	0.9300	C59—C60	1.367 (4)
C16—C17	1.376 (4)	C59—H59	0.9300
C17—H17	0.9300	C60—C61	1.429 (4)
C18—C19	1.385 (3)	C60—H60	0.9300
C18—C27	1.435 (4)	C63—C64	1.382 (4)
C19—C20	1.407 (4)	C63—C68	1.400 (4)
C20—C21	1.358 (4)	C64—C65	1.388 (5)
C20—H20	0.9300	C64—H64	0.9300
C21—C22	1.409 (4)	C65—C66	1.368 (5)
C21—H21	0.9300	C66—C67	1.375 (5)
C22—C23	1.423 (4)	C66—H66	0.9300
C22—C27	1.426 (4)	C67—C68	1.396 (4)
C23—C24	1.347 (4)	C68—H68	0.9300
C23—H23	0.9300	Cl10—C100	1.729 (6)
C24—C25	1.400 (5)	Cl12—C100	1.693 (6)
C24—H24	0.9300	Cl11—C100	1.765 (6)
C25—C26	1.363 (4)	C100—H100	0.9800
C25—H25	0.9300	Cl13—C101	1.614 (9)
C26—C27	1.418 (4)	Cl14—C101	1.712 (9)
C26—H26	0.9300	Cl15—C101	1.759 (8)
C29—C30	1.386 (4)	C101—H101	0.9800
C29—C34	1.408 (4)	Cl20—C200	1.761 (6)
C30—C31	1.382 (4)	Cl21—C200	1.745 (8)
C30—H30	0.9300	Cl22—C200	1.754 (6)
C31—C32	1.383 (4)	C200—H200	0.9800
C32—C33	1.382 (5)	Cl23—C201	1.755 (10)
C32—H32	0.9300	Cl25—C201	1.747 (9)
C33—C34	1.366 (4)	Cl24—C201	1.741 (11)
C34—H34	0.9300	C201—H201	0.9800
Cl5—C48	1.737 (4)	Cl30—C300	1.724 (10)
Cl6—C50	1.730 (4)	Cl31—C300	1.742 (10)
Cl7—C65	1.738 (4)	Cl32—C300	1.736 (9)
Cl8—C67	1.744 (3)	C300—H300	0.9800
O3—C45	1.236 (3)	Cl33—C301	1.724 (11)
O4—C62	1.229 (3)	Cl35—C301	1.736 (10)
N5—C45	1.362 (3)	Cl34—C301	1.743 (11)
N5—C36	1.431 (4)	C301—H301	0.9800
N5—H5A	0.8600	Cl40—C400	1.743 (9)
N6—C45	1.369 (4)	Cl41—C400	1.691 (8)
N6—C46	1.408 (4)	Cl42—C400	1.687 (7)
N6—H6A	0.8600	C400—H400	0.9800
N7—C62	1.359 (4)	Cl45—C401	1.763 (16)
N7—C53	1.411 (3)	C401—Cl43	1.723 (15)
N7—H7A	0.8600	C401—Cl44	1.780 (15)
N8—C62	1.358 (3)	C401—H401	0.9800
			
C11—N1—C2	127.1 (3)	C38—C39—C44	120.2 (3)
C11—N1—H1A	116.4	C38—C39—C40	121.5 (2)
C2—N1—H1A	116.4	C44—C39—C40	118.3 (2)
C11—N2—C12	127.1 (2)	C41—C40—C39	121.0 (3)
C11—N2—H2A	116.4	C41—C40—H40	119.5
C12—N2—H2A	116.4	C39—C40—H40	119.5
C28—N3—C19	123.0 (2)	C40—C41—C42	119.9 (3)
C28—N3—H3A	118.5	C40—C41—H41	120.0
C19—N3—H3A	118.5	C42—C41—H41	120.0
C28—N4—C29	126.0 (2)	C43—C42—C41	121.6 (3)
C28—N4—H4A	117.0	C43—C42—H42	119.2
C29—N4—H4A	117.0	C41—C42—H42	119.2
C2—C1—C10	119.2 (2)	C42—C43—C44	120.2 (3)
C2—C1—C18	119.6 (2)	C42—C43—H43	119.9
C10—C1—C18	121.1 (2)	C44—C43—H43	119.9
C1—C2—C3	121.1 (3)	C43—C44—C39	119.1 (3)
C1—C2—N1	118.2 (2)	C43—C44—C35	122.6 (2)
C3—C2—N1	120.6 (2)	C39—C44—C35	118.3 (2)
C4—C3—C2	119.8 (3)	O3—C45—N5	122.8 (3)
C4—C3—H3	120.1	O3—C45—N6	123.4 (2)
C2—C3—H3	120.1	N5—C45—N6	113.8 (2)
C3—C4—C5	121.6 (3)	C51—C46—C47	119.9 (3)
C3—C4—H4	119.2	C51—C46—N6	118.8 (3)
C5—C4—H4	119.2	C47—C46—N6	121.3 (3)
C4—C5—C6	122.6 (3)	C48—C47—C46	118.4 (3)
C4—C5—C10	119.1 (3)	C48—C47—H47	120.8
C6—C5—C10	118.2 (3)	C46—C47—H47	120.8
C7—C6—C5	121.6 (3)	C49—C48—C47	122.9 (3)
C7—C6—H6	119.2	C49—C48—Cl5	118.7 (3)
C5—C6—H6	119.2	C47—C48—Cl5	118.4 (3)
C6—C7—C8	120.0 (3)	C48—C49—C50	117.0 (3)
C6—C7—H7	120.0	C48—C49—H49	121.5
C8—C7—H7	120.0	C50—C49—H49	121.5
C9—C8—C7	120.6 (3)	C51—C50—C49	121.8 (3)
C9—C8—H8	119.7	C51—C50—Cl6	119.6 (3)
C7—C8—H8	119.7	C49—C50—Cl6	118.6 (3)
C8—C9—C10	121.1 (3)	C46—C51—C50	119.9 (3)
C8—C9—H9	119.4	C46—C51—H51	120.1
C10—C9—H9	119.4	C50—C51—H51	120.1
C9—C10—C5	118.4 (3)	C53—C52—C61	119.1 (2)
C9—C10—C1	122.4 (3)	C53—C52—C35	119.4 (2)
C5—C10—C1	119.2 (3)	C61—C52—C35	121.4 (2)
O1—C11—N1	124.1 (3)	C52—C53—N7	117.5 (2)
O1—C11—N2	123.0 (3)	C52—C53—C54	120.7 (2)
N1—C11—N2	112.8 (3)	N7—C53—C54	121.6 (2)
C17—C12—C13	120.5 (3)	C55—C54—C53	119.7 (3)
C17—C12—N2	117.5 (3)	C55—C54—H54	120.2
C13—C12—N2	121.8 (3)	C53—C54—H54	120.2
C14—C13—C12	118.1 (3)	C54—C55—C56	122.3 (2)
C14—C13—H13	120.9	C54—C55—H55	118.9
C12—C13—H13	120.9	C56—C55—H55	118.9
C13—C14—C15	123.5 (3)	C57—C56—C55	122.6 (3)
C13—C14—Cl2	118.7 (2)	C57—C56—C61	119.6 (3)
C15—C14—Cl2	117.8 (2)	C55—C56—C61	117.8 (2)
C14—C15—C16	116.1 (3)	C58—C57—C56	121.1 (3)
C14—C15—H15	121.9	C58—C57—H57	119.5
C16—C15—H15	121.9	C56—C57—H57	119.5
C17—C16—C15	122.4 (3)	C57—C58—C59	119.4 (3)
C17—C16—Cl1	119.5 (2)	C57—C58—H58	120.3
C15—C16—Cl1	118.1 (2)	C59—C58—H58	120.3
C16—C17—C12	119.4 (3)	C60—C59—C58	121.6 (3)
C16—C17—H17	120.3	C60—C59—H59	119.2
C12—C17—H17	120.3	C58—C59—H59	119.2
C19—C18—C27	118.6 (2)	C59—C60—C61	120.2 (3)
C19—C18—C1	120.5 (2)	C59—C60—H60	119.9
C27—C18—C1	120.7 (2)	C61—C60—H60	119.9
C18—C19—C20	121.7 (3)	C52—C61—C56	120.2 (2)
C18—C19—N3	119.2 (2)	C52—C61—C60	121.8 (2)
C20—C19—N3	119.1 (2)	C56—C61—C60	118.0 (2)
C21—C20—C19	119.9 (2)	O4—C62—N8	123.2 (2)
C21—C20—H20	120.0	O4—C62—N7	123.7 (2)
C19—C20—H20	120.0	N8—C62—N7	113.1 (2)
C20—C21—C22	121.4 (3)	C64—C63—C68	120.9 (3)
C20—C21—H21	119.3	C64—C63—N8	119.9 (3)
C22—C21—H21	119.3	C68—C63—N8	119.2 (3)
C21—C22—C23	122.1 (3)	C63—C64—C65	118.8 (3)
C21—C22—C27	119.1 (3)	C63—C64—H64	120.6
C23—C22—C27	118.8 (2)	C65—C64—H64	120.6
C24—C23—C22	120.9 (3)	C66—C65—C64	122.3 (3)
C24—C23—H23	119.5	C66—C65—Cl7	119.0 (3)
C22—C23—H23	119.5	C64—C65—Cl7	118.7 (3)
C23—C24—C25	120.4 (3)	C65—C66—C67	118.0 (3)
C23—C24—H24	119.8	C65—C66—H66	121.0
C25—C24—H24	119.8	C67—C66—H66	121.0
C26—C25—C24	121.1 (3)	C66—C67—C68	122.6 (3)
C26—C25—H25	119.5	C66—C67—Cl8	119.6 (2)
C24—C25—H25	119.5	C68—C67—Cl8	117.8 (3)
C25—C26—C27	120.3 (3)	C67—C68—C63	117.5 (3)
C25—C26—H26	119.8	C67—C68—H68	121.3
C27—C26—H26	119.8	C63—C68—H68	121.3
C26—C27—C22	118.5 (3)	Cl12—C100—Cl10	117.4 (3)
C26—C27—C18	122.3 (3)	Cl12—C100—Cl11	112.0 (4)
C22—C27—C18	119.2 (2)	Cl10—C100—Cl11	112.6 (3)
O2—C28—N3	123.1 (2)	Cl12—C100—H100	104.4
O2—C28—N4	123.1 (2)	Cl10—C100—H100	104.4
N3—C28—N4	113.8 (2)	Cl11—C100—H100	104.4
C30—C29—C34	120.1 (3)	Cl13—C101—Cl14	121.0 (5)
C30—C29—N4	122.7 (2)	Cl13—C101—Cl15	113.7 (5)
C34—C29—N4	117.3 (2)	Cl14—C101—Cl15	111.0 (5)
C31—C30—C29	118.3 (3)	Cl13—C101—H101	102.8
C31—C30—H30	120.8	Cl14—C101—H101	102.8
C29—C30—H30	120.8	Cl15—C101—H101	102.8
C30—C31—C32	123.3 (3)	Cl21—C200—Cl22	112.0 (4)
C30—C31—Cl3	117.3 (2)	Cl21—C200—Cl20	109.4 (4)
C32—C31—Cl3	119.4 (2)	Cl22—C200—Cl20	111.8 (4)
C33—C32—C31	116.3 (3)	Cl21—C200—H200	107.8
C33—C32—H32	121.9	Cl22—C200—H200	107.8
C31—C32—H32	121.9	Cl20—C200—H200	107.8
C34—C33—C32	123.4 (3)	Cl24—C201—Cl25	109.4 (5)
C34—C33—Cl4	118.4 (2)	Cl24—C201—Cl23	111.8 (6)
C32—C33—Cl4	118.3 (2)	Cl25—C201—Cl23	109.6 (6)
C33—C34—C29	118.5 (3)	Cl24—C201—H201	108.6
C33—C34—H34	120.7	Cl25—C201—H201	108.6
C29—C34—H34	120.7	Cl23—C201—H201	108.6
C45—N5—C36	120.5 (2)	Cl30—C300—Cl32	111.5 (5)
C45—N5—H5A	119.7	Cl30—C300—Cl31	110.9 (6)
C36—N5—H5A	119.7	Cl32—C300—Cl31	110.4 (5)
C45—N6—C46	125.3 (2)	Cl30—C300—H300	108.0
C45—N6—H6A	117.3	Cl32—C300—H300	108.0
C46—N6—H6A	117.3	Cl31—C300—H300	108.0
C62—N7—C53	127.2 (2)	Cl33—C301—Cl35	112.2 (6)
C62—N7—H7A	116.4	Cl33—C301—Cl34	110.1 (7)
C53—N7—H7A	116.4	Cl35—C301—Cl34	109.8 (6)
C62—N8—C63	122.9 (2)	Cl33—C301—H301	108.2
C62—N8—H8A	118.5	Cl35—C301—H301	108.2
C63—N8—H8A	118.5	Cl34—C301—H301	108.2
C36—C35—C44	119.5 (2)	Cl42—C400—Cl41	116.4 (5)
C36—C35—C52	121.7 (2)	Cl42—C400—Cl40	112.7 (5)
C44—C35—C52	118.8 (2)	Cl41—C400—Cl40	113.6 (5)
C35—C36—C37	121.2 (3)	Cl42—C400—H400	104.1
C35—C36—N5	120.4 (2)	Cl41—C400—H400	104.1
C37—C36—N5	118.3 (2)	Cl40—C400—H400	104.1
C38—C37—C36	120.6 (2)	Cl43—C401—Cl45	113.6 (11)
C38—C37—H37	119.7	Cl43—C401—Cl44	103.4 (9)
C36—C37—H37	119.7	Cl45—C401—Cl44	114.7 (11)
C37—C38—C39	120.2 (2)	Cl43—C401—H401	108.3
C37—C38—H38	119.9	Cl45—C401—H401	108.3
C39—C38—H38	119.9	Cl44—C401—H401	108.3

### Hydrogen-bond geometry (Å, °)

**Table:**

Cg1 is the centroid of the C56–C61 ring.

**Table:**

*D*—H···*A*	*D*—H	H···*A*	*D*···*A*	*D*—H···*A*
N3—H3A···O4^i^	0.86	2.16	2.897 (3)	144
N4—H4A···O4^i^	0.86	2.05	2.821 (3)	149
N5—H5A···O2	0.86	2.11	2.821 (3)	139
N6—H6A···O2	0.86	2.17	2.947 (3)	150
N7—H7A···O3	0.86	2.36	2.955 (3)	126
N8—H8A···O3	0.86	2.26	3.030 (3)	149
C3—H3···O1	0.93	2.44	2.937 (3)	113
C13—H13···O1	0.93	2.47	2.943 (3)	112
C30—H30···O2	0.93	2.38	2.864 (3)	113
C38—H38···O1^ii^	0.93	2.41	3.304 (4)	162
C47—H47···O3	0.93	2.50	2.911 (4)	107
C54—H54···O4	0.93	2.32	2.893 (3)	120
C200—H200···O1^ii^	0.98	2.15	3.035 (7)	149
C300—H300···O3	0.98	2.52	3.315 (9)	138
N2—H2A···Cg1	0.86	2.59	3.300 (3)	141

Symmetry codes: (i) *x*, *y*+1, *z*; (ii) *x*+1, *y*, *z*.
